# Homology-based loop modeling yields more complete crystallographic protein structures

**DOI:** 10.1107/S2052252518010552

**Published:** 2018-08-08

**Authors:** Bart van Beusekom, Krista Joosten, Maarten L. Hekkelman, Robbie P. Joosten, Anastassis Perrakis

**Affiliations:** a Department of Biochemistry, The Netherlands Cancer Institute, Plesmanlaan 121, Amsterdam 1066CX, The Netherlands

**Keywords:** loop building, structural re-building, PDB-REDO, model completion, crystallography

## Abstract

Inherent protein flexibility, poor or low-resolution diffraction data or poorly defined electron-density maps often inhibit the building of complete structural models during X-ray structure determination. However, recent advances in crystallographic refinement and model building often allow completion of previously missing parts. This paper presents algorithms that identify regions missing in a certain model but present in homologous structures in the Protein Data Bank (PDB), and ‘graft’ these regions of interest. These new regions are refined and validated in a fully automated procedure. Including these developments in the *PDB-REDO* pipeline has enabled the building of 24 962 missing loops in the PDB. The models and the automated procedures are publicly available through the PDB-REDO databank and webserver. More complete protein structure models enable a higher quality public archive but also a better understanding of protein function, better comparison between homologous structures and more complete data mining in structural bioinformatics projects.

## Abstract 2

Thousands of regions missing from existing protein structure models are completed using new methods based on homology.

## 1. Introduction   

Protein structure models give direct and detailed insights into biochemistry (Lamb *et al.*, 2015 ▸) and are therefore highly relevant to many areas of biology and biotechnology (Terwilliger & Bricogne, 2014 ▸). For decades, crystallography has been the leading technique in determining protein structure models (Berman *et al.*, 2014 ▸) and to date, over 120 000 crystallographic structure models are available from the Protein Data Bank (PDB; Burley *et al.*, 2017 ▸). It is important to realize that all structures are interpretations of the underlying experimental data (Lamb *et al.*, 2015 ▸; Wlodawer *et al.*, 2013 ▸) and the quality of a structure model should therefore be scrutinized by validation (Read *et al.*, 2011 ▸; Richardson *et al.*, 2013 ▸).

**Figure 1:**
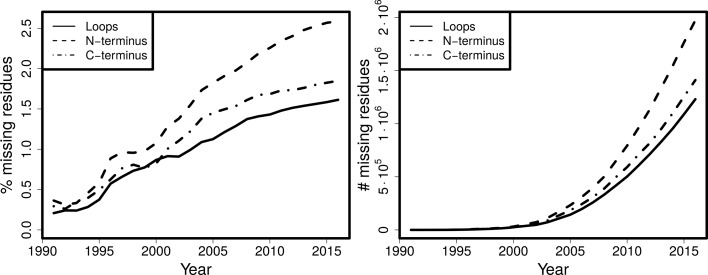
Cumulative percentage (left) and absolute number (right) of residues missing in termini and in loops for all structures over the years.

Owing to numerous improvements in refinement and validation methods, the quality of protein structure models is continuously increasing (Read *et al.*, 2011 ▸); however, the completeness of models is decreasing (Fig. 1 ▸). About 70% of all crystallographic protein structures have regions that are missing (Djinovic-Carugo & Carugo, 2015 ▸) and this percentage is increasing. Typically, but not necessarily, these missing regions are loops between helices and strands. Loops occupy a large conformational space and can therefore be missing as a result of intrinsic disorder, meaning they cannot be modeled reliably in a single conformation. However, there are many cases where the experimental data provide useful information on the loop conformation and hence many loops can be built into protein structure models. The term ‘loop’ will be used in this paper in its broader definition to denote a missing region of protein structure, regardless of secondary structure conformation.

Missing protein regions or ‘loops’ are typically modeled towards the end of crystal structure determination. By that stage, all obvious features have typically been modeled, the electron-density maps have improved and it is possible to model the loops. Many programs are available for modeling loops either interactively (Emsley *et al.*, 2010 ▸) or automatically (Terwilliger *et al.*, 2008 ▸; Joosten *et al.*, 2008 ▸; Cowtan, 2012 ▸; Kleywegt & Jones, 1998 ▸; DePristo *et al.*, 2005 ▸). Completing the protein structure by modeling all loops that can be modeled, has two advantages: locally, the density becomes unavailable for modeling erroneous structural features such as other parts of the protein (*e.g.* side chains), crystallization agents and water molecules; and globally, a correctly fitted loop will reduce the phase error and give an overall improvement of the electron-density maps. Available loop-building approaches rely on one form or another of conformational sampling that attempts to find the best-fitting conformation of the loops based on the local electron density and our general knowledge of protein structure.

Building loops is often one of the most difficult, time-demanding and sometimes frustrating stages of crystallographic model building. If loops are too disordered to yield traceable electron density, they cannot be built and there is no problem. In many cases however, loops are sufficiently rigid to yield interpretable density. Typically, this electron density is not as clear as desired, which makes it challenging for crystallographers and model-building programs to model a loop in a realistic conformation. Whether this is eventually successful depends on many factors such as perseverance and skill of the crystallographer(s) and the algorithmic quality and ease-of-use of loop-building programs. Noteworthy are algorithms that attempt to interpret the electron density with multiple loop conformations (Burnley *et al.*, 2012 ▸; Levin *et al.*, 2007 ▸). Although there is little doubt that very often multiple conformations can better represent the experimental data, here we wanted to deal with the problem where a loop is not modeled at all despite strong experimental evidence that it can be modeled.

Previously, we have developed algorithms to transfer information about homologous protein structures for obtaining geometric restraints for low-resolution refinement (van Beusekom *et al.*, 2018 ▸). Here we exploit the relationship between homologous proteins for loop building. We reasoned that as highly similar (in terms of sequence) proteins have very similar structure, the conformation of a loop in one homologous structure can assist in identifying the loop location in another homologous structure that is being built. The presence of a loop in a homologous structure is also an indication that loop building is viable. If a region has not been modeled in any of the homologs, it is unlikely to be buildable in the new structure (provided there are many homologous models solved by different crystallographers), but if it is built at least once there is a good chance that the same region can be modeled in the structure in question. Of course there are exceptions to the similarity of homologous loops, as loops can adopt distinct conformations which are often of high significance for function: there are countless examples that describe loop motions associated with ligand interactions or a change in the context of different crystallographic contacts.

It has already been shown for a set of 16 370 PDB structures (Le Gall *et al.*, 2007 ▸) that although 92.2% of all crystallized residues were always ordered in all homologs and 4.4% were always disordered, 3.3% are ‘ambiguous’; these residues were modeled in at least one structure but disordered in other(s). Another survey (Zhang *et al.*, 2007 ▸) observed that such regions, named ‘dual personality’ fragments, occur in 45% of sequence-identical structure groups. Of the ambiguous loop regions, 59% are predicted to be ordered by all three protein disorder predictors used in a third study (DeForte & Uversky, 2016 ▸). Thus, the increasing redundancy of homologs in the PDB, the increasing percentage of unmodeled residues (Fig. 1 ▸) and the disorder predictions for these regions, argue that using ‘dual personality’ regions between homologous structures is a viable strategy for increasing the completeness of protein structure models.

We have therefore decided to develop a procedure for building loops in cases where a loop is available in other homologous structure(s). The implementation of these new ideas has taken place in the context of *PDB-REDO*, a procedure we are developing to (re-)refine and partially rebuild protein structure models, both retrospectively [by updating existing PDB entries (Joosten *et al.*, 2012 ▸)] and proactively [our software is available as a webserver (Joosten *et al.*, 2014 ▸) and for local installation]. As the PDB-REDO electron-density maps are often better than the original maps and can be simply recalculated from the PDB entries, incorporating this work in the PDB-REDO framework allows us to have the best possible maps for building and validation of the missing loops. For every structure in question, the missing loop is first identified, then built by grafting it from a homologous structure, refined to fit the electron-density map in real space, and finally validated against geometric criteria and the electron density. We have built and validated several thousands of loops missing from structures deposited in the PDB. Here we discuss the methods and show some examples where our procedure makes a notable difference in the structure model.

## 2. Methods   

### 2.1. Loop building   

**Figure 2:**
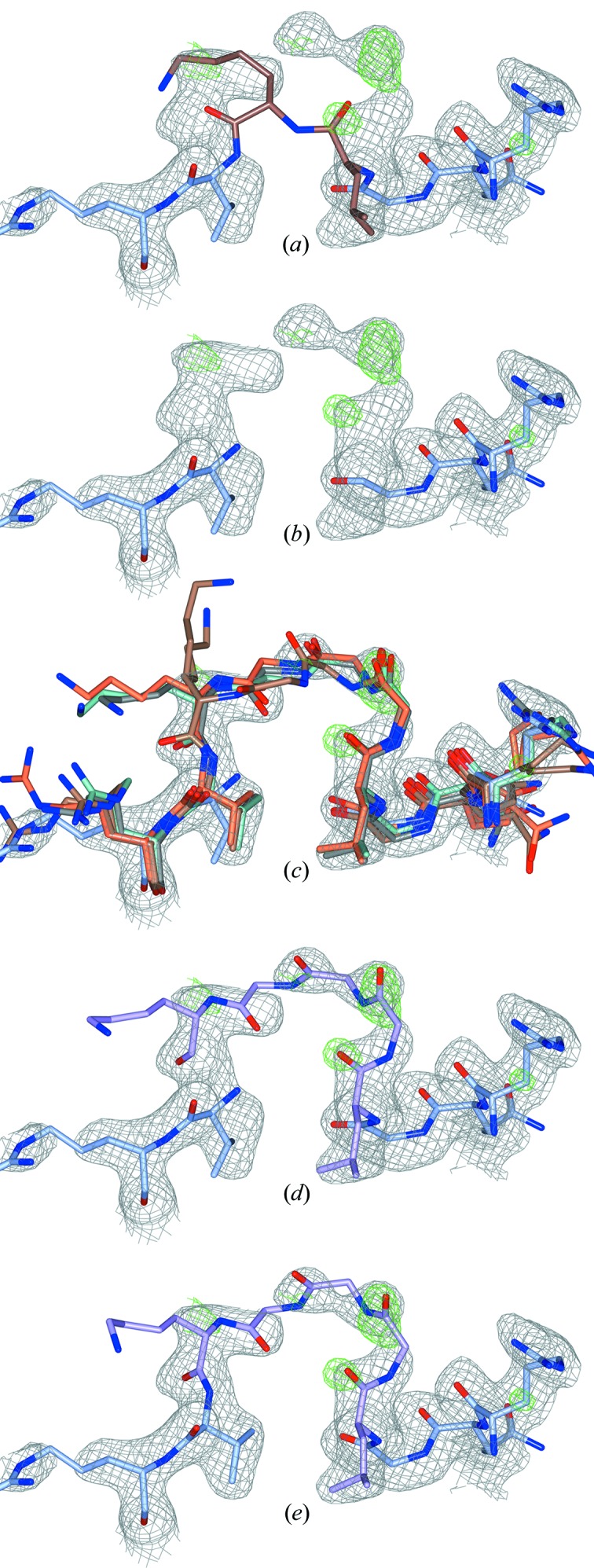
Stepwise illustration of the *Loopwhole* algorithm, applied to three missing glycines (PDB entry 1dmn; Kim *et al.*, 2000 ▸). All 2*mF*_o_ − *DF*_c_ and *mF*_o_ − *DF*_c_ electron-density maps are shown at 0.7σ and 3.0σ, respectively. (*a*) The PDB structure has no apparent gap: two residues are wrongly bound to one another (highlighted in brown). The gap is detected from the sequence. (*b*) The two residues immediately adjacent to the missing loop are removed, as these are often in the wrong conformation. (*c*) All homologous chains with the loop present are structurally aligned to the target structure model (only four homologs shown). (*d*) If the surrounding residues align well, the loop is grafted into the target structure model. By default, the top ten alignments are kept (only one shown). (*e*) After real-space optimization, the loop with the best density fit is kept provided the density fit and geometrical quality pass the filter criteria.

We have developed algorithms to transfer loops from homologous protein structures to the target structure (Fig. 2 ▸). The manner of handling the homologs in *Loopwhole* is nearly identical to our previous program *HODER* (Beusekom *et al.*, 2018 ▸), which generates homology-based hydrogen-bond restraints. The only difference is that homologs are not filtered by resolution in *Loopwhole*. The default maximum length for attempted loop transfer is 30 amino acids.

Before any loop building is attempted, the density fit per chain is evaluated with *EDSTATS* (Tickle, 2012 ▸). In the rare case that the real-space correlation coefficient (RSCC) for an entire chain is below 0.80, we do not attempt to build loops in that chain, but instead warn the user that the density fit is low making the overall chain conformation unreliable.

There are two initial requirements for loop transfer: the presence of unmodeled loops in the input structure and modeled equivalent loops in the homologs. Unmodeled loops are detected using *pdb2fasta* in *PDB-REDO* and high-identity homologs are aligned by sequence. The details of these algorithms have been described earlier (van Beusekom *et al.*, 2018 ▸). If both requirements are met, loop transfer is attempted for each of the homologs that has a complete backbone model for that homolog. Both input model and homolog are required to have at least five consecutive modeled residues on each side of the loop. Of these ten residues, the two residues directly adjacent to the loop are remodeled together with the loop because they were often found to be in a suboptimal conformation. The remaining eight residues are used for alignment. To prepare residues for the alignment, side-chain atoms are deleted when mutations are present, and administrative flips for Asp, Glu, Phe and Tyr residues (DEFY flips) are performed to ensure equivalent atoms are in equivalent positions. A homolog is skipped if the sequence identity in the loop or the aligned residues is less than 50%; the exception is a single-residue loop, which is allowed to be mutated. Finally, structural alignment is performed using quaternions (Kuipers, 2002 ▸).

If the backbone RMSD of alignment is less than 2.0 Å (in default settings), an initial loop transfer is performed. The two residues directly adjacent to the loop are deleted and the aligned loop, including the two aligned and directly adjacent residues, is inserted into the protein model. In the transferred loop, side chains are cropped where appropriate in the case of mutations, the occupancy of all atoms is set to 1.00 and the *B* factor is multiplied for each atom by the ratio of average *B* factors between input structure and homolog. Me­thio­nine is mutated to seleno­methio­nine and *vice versa* based on the other residues present in the input model.

The next check for the initial transfer of a loop is the evaluation of clashes with the modeled atoms already present, or with symmetry copies thereof. We make a distinction between main-chain and side-chain atom clashes. Since the position of a C^β^ is significantly limited by the main-chain conformation, we include this side-chain atom in the main chain for clash analysis. Heavy clashes are defined as atom–atom distances <2.1 Å and small clashes as <2.6 Å.

In cases of clashes, important atom(s) must be retained. In *Loopwhole* we use hierarchical rules of atom importance to decide how to proceed. The atoms that are always kept are main-chain atoms and most ligands [the exceptions are glycerol, ethanol, and 1,2-ethane­diol and its polymeric (PEG) forms]. Whenever a main-chain atom from a loop candidate clashes with the previously modeled backbone or most ligands, the loop candidate is discarded. In contrast, if the main chain of a loop candidate clashes with a compound from the list of exceptions (such as glycerol), that compound is discarded. The second most important group is the side chains: they can be discarded temporarily to be added back later by the program *SideAide* (Joosten *et al.*, 2011 ▸). Previously modeled side chains are considered more important than loop side chains. Side chains, in turn, are considered more important than water molecules and any atoms with an occupancy of 0.01 or lower. These principles led to the following decisions.

Previously modeled side chains are removed only if they clash heavily with the loop backbone, unless they form a cysteine bridge: in such cases the loop candidate is discarded. Loop side chains [from γ-atom(s) onwards] are deleted if they clash heavily with any previously modeled protein (main chain or side chain) or with any other compound except for water. Ligands from the exception list are removed whenever they clash heavily with the main chain of the loop. Waters and atoms with an occupancy of 0.01 or less are removed even in cases of small clashes with any loop atom.

If there are no insuperable clashes, the loop candidate is saved and existing candidates are sorted according to RMSD. If two loop candidates have a very similar conformation (RMSD < 0.1 Å), the candidate with the worst RMSD of alignment is discarded. Once all *BLAST* hits are evaluated, the top candidates (by default, the top ten) are subjected to real-space refinement by *coot-mini-rsr* (Emsley *et al.*, 2010 ▸) using torsion-angle restraints. One extra residue from the existing protein on either side of the loop is added to the real-space refinement region, which allows the existing protein model to better adapt to the new loop. In the *coot-mini-rsr* input PDB file, clashing atoms are removed, atom numbering is updated (including CONECT records) and ‘gap’ LINK records are deleted. Sometimes, there are still small gaps at the boundary of the transferred loop and the existing model. To increase the success rate of *coot-mini-rsr* closing these gaps, the backbone N atoms on the loop edges are moved into this gap. This can temporarily create unlikely atom bond lengths and angles, but these will be resolved in real-space refinement (or in the subsequent reciprocal-space refinement).

After running *coot-mini-rsr*, it is checked again to confirm whether there are no insuperable clashes between the loop and the protein, because we have observed that the loop may be placed into the density of other moieties in the real-space refinement, such as a symmetry copy of itself.

At this stage, all remaining loop candidates with bad geometry are discarded. First, candidates where there is no peptide bond between two consecutive residues or where *coot-mini-rsr* has not converged to a minimum are removed. The resulting RMS *Z* scores from *coot-mini-rsr* are used to filter bad geometry candidates: loops are rejected if bond or angle RMS *Z* values exceed 1.2, chirality RMS *Z* exceeds 1.5, or if plane or torsion RMS *Z* values exceed 2.0. In this filter, the RMS *Z* values are allowed to be relatively high because subsequent reciprocal-space refinement will further improve the loop. Loop candidates are only allowed to have *cis*-peptides if the corresponding residue in the original loop of the homolog is also a *cis*-peptide. Loops that have multiple sequential distorted omega angles (maximum deviation 30° from 0 or 180°) are discarded, but single distortions are allowed as these are usually resolved in subsequent refinement. Finally, loop candidates are evaluated on their Ramachandran *Z* score. If the *Z* score is poor (lower than −5), it is compared with the *Z* scores of the other loop candidates and also the *Z* scores of the loop in the homologous-structure models from which it was adapted. Then, the candidate is discarded if it is a 2σ negative outlier [according to Grubbs’ test (Grubbs, 1950 ▸)], either compared with the other loop candidates or with the original conformation of the loop. The Ramachandran *Z*-score calculation is performed using the algorithms of the new *PDB-REDO* program *tortoize*. This algorithm is based on the implementation in *WHAT_CHECK* (Hooft *et al.*, 1997 ▸) and is described in the Supporting Information.

The density for each remaining candidate is then computed using the cubic interpolation function from *clipper* (Cowtan, 2003 ▸). It is computed only for the main-chain atoms of the candidate loop to ensure that the metric is not influenced by the presence, absence or length of the side chains of the loop. Additionally, the density is computed for all main-chain atoms that are ordered in all homologs, *i.e.* the set of atoms that are always ordered. If there are fewer than 30 atoms in this set, all non-loop main-chain atoms in the input structure model are taken. The ratio between average loop-candidate density and the average density of the control set is then computed. This ratio must be over 0.25 for a loop candidate to be acceptable. The cut-off was established after manual inspection of several hundred candidate loops.

Finally, there is an option to subject a number of candidates (by default only the loop with the best density fit) to the *PDB-REDO* programs *SideAide* and *pepflip* (Joosten *et al.*, 2011 ▸) to complete the side chains and check for potential peptide flips of the loop area. However, this is not default behavior since these programs are already run after *Loopwhole* in the *PDB-REDO* implementation. However, *Loopwhole* writes a list of amino acids whose side chains are incomplete: at low resolution, *SideAide* is not run by default on all amino acids in *PDB-REDO*, but only on amino acids in a list, to which the novel residues in the loop are added.

After the optional running of *SideAide* and *pepflip*, the loop with the best main-chain density fit is kept.

There are a few special cases where detection or building of a missing loop is more complicated. First, there is the possibility that a loop is in fact modeled, but with all atoms modeled at zero occupancy or at an occupancy of 0.01. We consider such loops as unmodeled and proceed as described above; however, we treat the current zero-occupancy loop itself as an extra candidate. Since this loop is already at the correct location in the model, no alignment is necessary; aside from this, the candidate is treated the same as others.

Another special case is dealing with alternates in and near loops. If there are any main-chain alternates among the residues that are to be aligned with homologs, the missing loop is skipped because the alignment target is ambiguous. An exception is made if the only backbone alternate atoms are C^α^ atoms (which is common for residues with alternate side-chain conformations); then simply the first atom is picked. In such cases, the positions of alternate C^α^ atoms are very close to each other. Alternate side chains are truncated before alignment. In homologs, backbone alternate conformations are treated as separate candidates: structure alignment is performed for each alternate in the homolog and/or each alternate loop is transplanted. If there are multiple stretches that contain alternates with full occupancy atoms in between, combinations of these alternates must be aligned for completeness and the exponential increase of combinations makes computation expensive, hence these (rare) cases are excluded.

Finally, there are cases where residues next to a loop are only partially present. In such cases, the partially modeled residue is also removed before the loop fitting. That is, the loop is extended by one more residue and the partial residue is replaced.

### 2.2. Adding missing atoms, atom pairs or atom trios: *fixDMC*   

We observed that some protein models are missing one or several atoms from a peptide backbone. In order to also correct these smaller missing parts, the program *fixDMC (fix ‘dat’ main chain)* corrects these omissions, adds missing C-terminal O atoms, and resets occupancies to 1.0 in regions where there are no alternates and surrounding atoms are modeled at full occupancy.

We make use of the fact that C^α*i*^, C^*i*^, O^*i*^, N^*i*^ and C^α*i*+1^ lie in a plane. Whenever at least three atoms of a single plane are present, this planarity combined with the known geometry of an amino acid gives enough information to compute its coordinates. The C^α^ atom lies in two planes: that of the preceding and the following amino-acid residues. Therefore, it can be added based on either of these residues. By applying the geometrical rules of planarity extensively, we can compute any set of one, two or three atoms provided that the preceding and following residues are modeled.

Additionally, *fixDMC* uses functionality from *pdb2fasta* in *PDB-REDO* (van Beusekom *et al.*, 2018 ▸) to add the second C-terminal O (‘OXT’) if the SEQRES records or user-inputted FASTA file indicate that the complete C-terminal residue has been modeled except for this atom. The addition of this atom can also be based on the peptide plane.

Finally, the occupancy of protein atoms is reset to full occupancy if the residue contains no alternates, and the preceding and subsequent atoms are both modeled at full occupancy. An exception is made for the carbonyl O atom: since this atom is only bound to a single C atom, only that C atom is required to be modeled at full occupancy.

### 2.3. Implementation in *PDB-REDO*   

The program *fixDMC* is run at the early stages of *PDB-REDO* after the initial electron-density maps are calculated, before any individual atomic coordinate or *B* factor refinement. The OXT atoms are only added if you can rely on the fact that the final modeled residue is the actual C-terminus of the crystallized construct. Therefore, this step is only performed if the header of the input PDB file has SEQRES records or if user-supplied sequence(s) can be mapped to the modeled atomic coordinates.

*Loopwhole* is run after the initial refinement in *PDB-REDO*. The default behavior is to always attempt to build loops, but this can be switched off if needed. It should be noted that on the PDB-REDO webserver, loops can only be built if the sequence of the missing residues is known. That is, users must supply the sequence as a FASTA file or as SEQRES records in the PDB file. If *Loopwhole* builds any residues, *REFMAC5* (Murshudov *et al.*, 2011 ▸) is run to obtain more accurate *B* factor estimates and new electron-density map coefficients. This refinement uses automated geometric restraint weighting and five refinement cycles; if a loop has an RSCC below 0.60, it is discarded and any water molecules or other compounds initially deleted to fit the loop are restored. Then the other rebuilding stages of *PDB-REDO* (Joosten *et al.*, 2011 ▸) are run.

Sequence files in *PDB-REDO* mark residues with a complete backbone in uppercase letters and incomplete or unmodeled residues with lowercase letters (van Beusekom *et al.*, 2018 ▸), an idea adopted from the SEQATOMS server (Brandt *et al.*, 2008 ▸). Therefore, both *Loopwhole* and *fixDMC* write updated FASTA files to reflect changes in residue completeness or presence. Additionally, *Loopwhole* updates the TLS groups in *PDB-REDO*. If a TLS group is surrounding the loop, the loop is added to that group; if the loop is on the border of two TLS groups, it is added to the first one.

At the final stage of *PDB-REDO*, the program *Modelcompare* writes a datafile that is used by *Coot* (Emsley *et al.*, 2010 ▸) and *3Dbionotes* (Segura *et al.*, 2017 ▸; Tabas-Madrid *et al.*, 2016 ▸) to highlight the new loops.

### 2.4. Testing   

*Loopwhole* was run over all entries available in PDB-REDO to identify which loops could be built. Hundreds of randomly selected loops were manually analyzed to empirically establish the validation cut-offs mentioned above. Finally, from all entries in which *Loopwhole* built loops, 2000 entries were randomly selected for further analysis in PDB-REDO. These entries were subjected to the *PDB-REDO* pipeline twice: once with and once without loop building. Owing to various limitations (not related to loop building), ten *PDB-REDO* jobs were not completed, hence the final test set consisted of 1990 entries.

## 3. Results   

### 3.1. Loop building   

**Figure 3:**
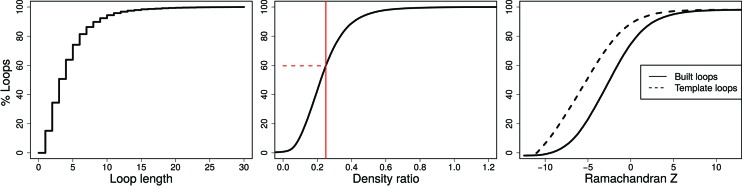
Cumulative distributions for properties of loops that can be built in the PDB. (Left) Most of the buildable loops are short. (Middle) Density ratio of loop candidates; in contrast to the other two subfigures, this includes loops that were not built. This metric represents the observed density for the loop main chain divided by the average main-chain density. The minimum required density ratio of 0.25 is indicated by the vertical red line. Of all initial loop candidates, 60% have insufficient density and are therefore discarded. (Right) The Ramachandran *Z* score for candidate loops and their counterparts in the structure model from which they were taken. The backbone conformation of the built loops is excellent and better than the conformation of loops in structures from which they were taken, which is largely a result of the application of Ramachandran restraints in the real-space refinement of loops in *coot-mini-rsr* (Emsley *et al.*, 2010 ▸).

The computer program *Loopwhole* was developed to build protein loops based on homology (Fig. 2 ▸). We first applied *Loopwhole* to the structures available in the PDB (Table 1 ▸). When *Loopwhole* was then applied to the PDB-REDO databank, we observed an increase of 11% in the number of built loops. This is likely to be because the structure models and the electron-density maps in PDB-REDO (which are obtained after modern re-refinement and rebuilding) are of higher quality than their ‘static’ PDB counterparts. The total number of missing loops in the PDB-REDO databank was 148 919. An initial loop was constructed by *Loopwhole* in 66 035 cases (44%). For the other 56%, there were either no homologous loops available, or the loop conformation was too different between the ‘donor’ and ‘acceptor’ structures as a result of genuine structural differences, or because of ‘sequence register’ errors. Another 41 073 loops (28% of total) were discarded according to various validation criteria (Fig. 3 ▸), keeping 24 962 successfully built loops in the final model. Many loops were rejected as their fit to the electron density was too poor (Fig. 3 ▸ *b*), and less often based on geometrical criteria or because both density fit and geometry were poor. The remaining loops have excellent geometry, typically better than the loop in the original structure (Fig. 3 ▸ *c*) and a good fit to the density.

The current version of *Loopwhole* was able to build a total of 24 962 missing loops in 11 571 entries. For 359 cases in which a loop was built, a zero-occupancy loop was present in the original model. To place the loops, 18 449 water molecules were removed; additionally, small molecules such as glycerol or ethane­diol were removed in 22 cases. The distribution of the length of the built loops is shown in Fig. 3 ▸(*a*).

**Figure 4:**
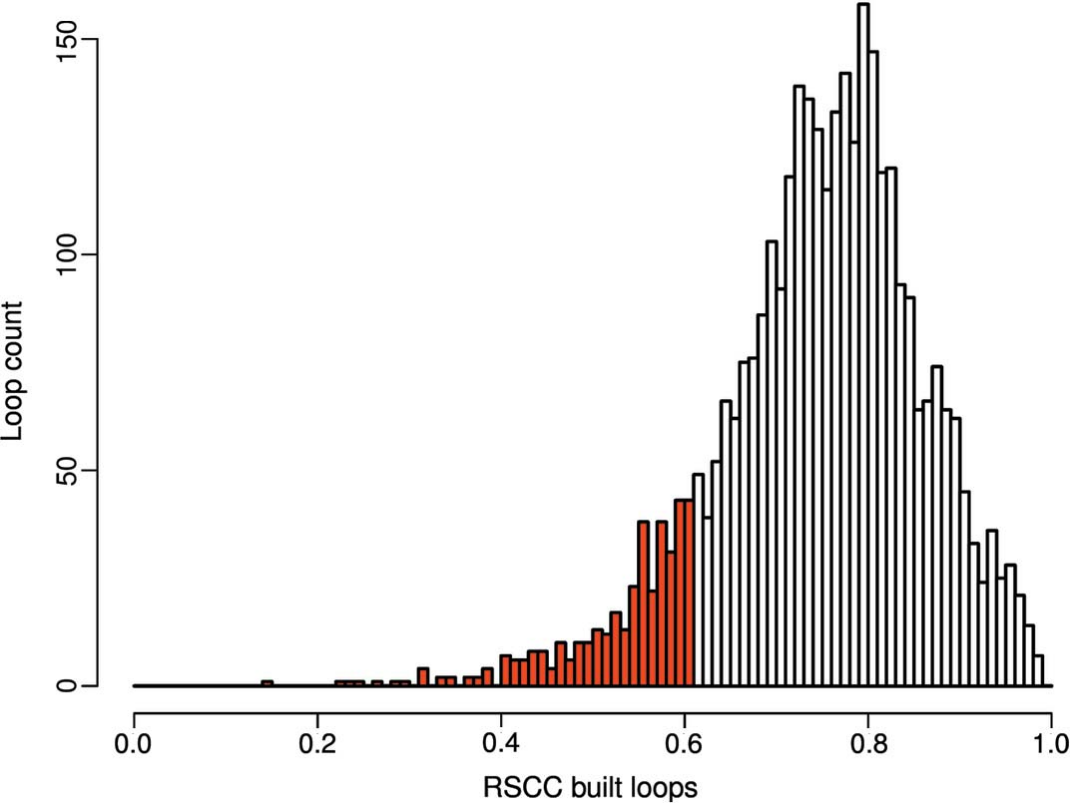
Distribution of the RSCC for all loops built in 1990 PDB-REDO entries. The RSCC was calculated on the final PDB-REDO structure models. All loops with an RSCC below 0.6 are colored red and are discarded.

**Figure 5:**
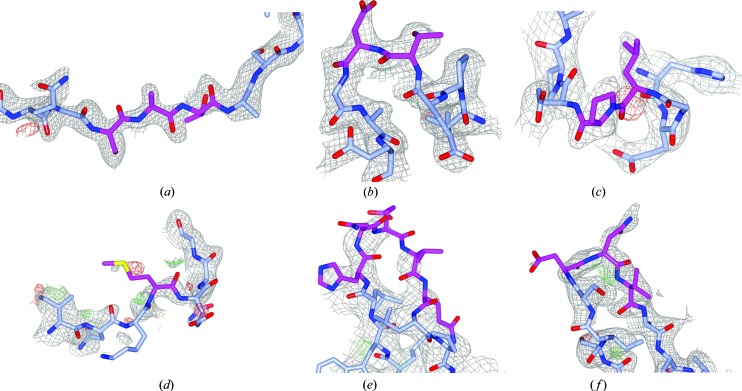
Six examples of loops with a density ratio between the cutoff value (0.25) and 0.30. The 2*mF*_o_ − *DF*_c_ maps are shown at 0.8σ; *mF*_o_ − *DF*_c_ maps are shown at 3.0σ. (*a*) 4fc9 C474–476, density ratio 0.27; (*b*) 5h8p A151-152, density ratio 0.29; (*c*) 2d31 A266-267, density ratio 0.25; (*d*) 2y1t F18, density ratio 0.27; (*e*) 2y7q B480–484, density ratio 0.26. For part of the loop, no density is observed at 0.8σ; however, it does show up at 0.6σ; (*f*) 4w9x A121–123, density ratio 0.26. For clarity, the side chain of TrpA124 is not shown.

Next, we examined whether incorporating *Loopwhole* in the *PDB-REDO* pipeline had an impact on the performance of *PDB-REDO* as a whole. We thus ran *PDB-REDO* on 1990 randomly selected structures in which loops could be built, once with and once without loop building. The impact of loop building on standard validation metrics (Read *et al.*, 2011 ▸) such as *R*_free_, the Ramachandran *Z* score and packing *Z* score was minimal (Fig. S1). The mean RSCC values (indicating the fit to the density) correlate well with the mean *B* factor for the loops in most cases (Fig. S2). The mean RSCC and RSR values for the loops themselves were 0.75 and 0.14, respectively (Fig. 4 ▸). These values are naturally lower than for well defined parts of the structure model, but are for example, consistent with density criteria for acceptable ligands (Weichenberger *et al.*, 2013 ▸; Cereto-Massagué *et al.*, 2013 ▸; Warren *et al.*, 2012 ▸). However, some built loops had lower than anticipated RSCC values. Following manual examination of example cases, we decided to discard loops with an RSCC below 0.60 (Fig. 4 ▸). Of the 3419 loops built in the test set, 305 loops were discarded in this step, yielding a total of 3114 loops built in the test entries. We then manually inspected examples of loops that passed the RSCC cutoff, but had density ratios between the cut-off value of 0.25 and 0.3. We concluded that these ‘lowest-quality’ loops still fit the density well and should be kept in the model; six randomly selected examples are shown in Fig. 5 ▸. Thus, loop building in *PDB-REDO* has now been enabled by default. For practical reasons (lack of CPU time) the remaining PDB entries are being gradually ‘redone’ and placed in the PDB-REDO databank.

### 3.2. Completing the main chain   

**Figure 6:**
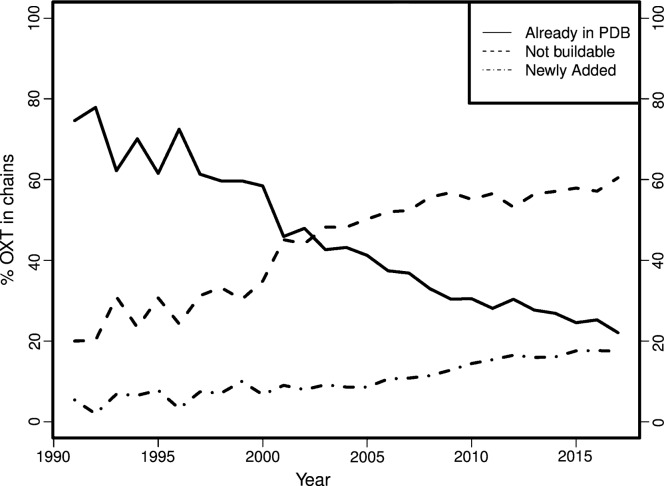
The percentage of C-terminal O atoms (OXT): present in the PDB; missing the C-terminal amino acid and thus not buildable; and newly added to the C-terminal amino acid.

Rather surprisingly, we found that many structures in the PDB are missing individual atoms in the main chain. Therefore, we created the program *fixDMC* that can add one to three missing main-chain atoms based on the geometry of the existing atoms (see §2 for details). Running it in the same PDB-REDO dataset as above, single atoms were added in 1500 cases (of which, 1281 were carbonyl O atoms), atom pairs were added in 55 cases and in 40 residues three atoms were added. Additionally, there were 38 101 cases with individual backbone atoms that had their occupancies set to values less than 1.00 without being part of an alternate conformation or of a partially occupied peptide ligand, out of which, 2926 cases had an occupancy of zero. Finally, we found that the second C-terminal O atom (OXT in PDB nomenclature) is missing in many PDB entries; *fixDMC* added OXTs to 41 120 protein chains, where the terminal residue in the structure coincides with the terminal residue in the declared construct sequence. Notably, the percentage of protein chains with missing C-terminal O atoms has been steadily increasing over the years (Fig. 6 ▸). In 2017, OXT is missing from 44% of chains with a modeled C-terminal amino acid.

### 3.3. Examples of built loops   

To illustrate the relevance of building loops in the PDB, here we show several examples in which *Loopwhole* clearly improves structure interpretation.

**Figure 7:**
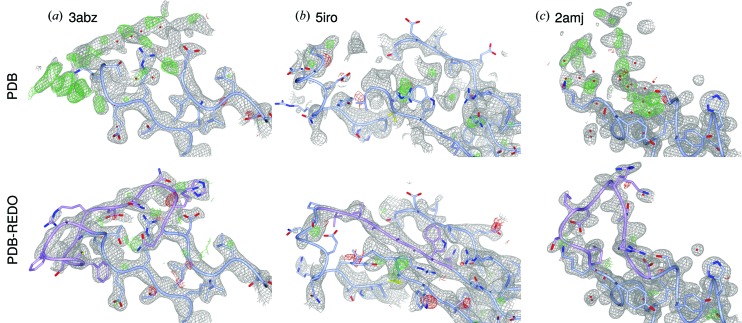
Examples of built loops. Newly built parts are shown in pink. (*a*) 3abz chain B residues 497–510. The RSCC for this loop is 0.76; in OMIT map it is 0.55. (*b*) 5iro chain I residues 243–249. RSCC values (normal/OMIT) are 0.84 and 0.98. (*c*) 2amj chain D residues 108–117. RSCC values (normal/OMIT) are 0.80 and 0.61. Details described in the main text. The 2*mF*_o_ − *DF*_c_ maps are shown at 0.8, 1.2 and 1.0σ, respectively. The *mF*_o_ − *DF*_c_ maps are shown at 3.0σ in all cases.

First, a structure of β-glucosidase (PDB entry 3abz; Yoshida *et al.*, 2010 ▸) has seven missing regions, five of which can be added. Only the first of four NCS copies is complete. One of the missing regions, a stretch of 14 residues, is shown in Fig. 7 ▸(*a*). The electron density for this loop is very clear. By adding the loops to the structure, the structure model is now much more complete and thus more easily interpreted.

A second example shows how the general improvement of fit to the crystallographic data in PDB-REDO can facilitate when loop building is included. As shown in Fig. 7 ▸(*b*), a strand is missing from the β-sheet of the α3 subunit of HLA in PDB entry 5iro (Li *et al.*, 2016 ▸), a protein complex of Adenovirus type 4 E3-19K with HLA class I histocompatibility antigen. By optimization of the structure model by *PDB-REDO* including loop building, the *R*/*R*_free_ drops from 0.220/0.236 to 0.182/0.190. The overall improvement of the model leads to much clearer maps into which the missing strand can be built unequivocally.

Finally, two loops are missing from PDB entry 2amj, the apo-structure model of modulator of drug activity B (MdaB), a putative DT-diaphorase (Adams & Jia, 2006 ▸). In this paper, the authors also discuss the FAD-bound state structure (2b3d; Adams & Jia, 2006 ▸), where both loops are ordered: it is stated, that these loops become ordered upon binding of FAD as a result of the rearrangement in the hydrogen-bonding network. However, there is clear density for one of the loops (L2). No less than 13 water molecules have been built into the density of the missing loop in chain D (Fig. 7 ▸ *c*). It was proposed (Adams & Jia, 2006 ▸) that FAD binding induces loop stabilization through changes in the hydrogen-bonding network; modeling this loop shows that the structure model of the apo-form and FAD-bound form are highly similar. Therefore, the structural evidence does not necessarily support this claim.

## 4. Discussion   

The increasing number of residues that are not built into new protein structures can be attributed to many factors: the ever larger structures determined by X-ray crystallography (van Beusekom *et al.*, 2016 ▸) are more likely to contain flexible regions within stable scaffolds; better annotation of the sequence of crystallized constructs (Henrick *et al.*, 2008 ▸; Dutta *et al.*, 2009 ▸) highlights missing regions better; and opportunities to built loops supported by the electron density are ignored because of haste or lack of experience (Mowbray *et al.*, 1999 ▸) as new generations of crystallographers are determining structures with a higher throughput. A worrying observation we made whilst teaching is that some students had deleted parts of a model to improve validation statistics (Read *et al.*, 2011 ▸) such as the percentage of RSR *Z* outliers.

It is generally accepted that loops with a less defined electron density occur in multiple conformations. In many cases, a single model with a sufficiently large margin of error represented in the *B* factors is suitable to represent the experimental data. There may be cases where more than one discrete loop could be modeled. At present in such cases, we build a single conformation for these loops. Though an extension towards building multiple conformations of such loops is possible, existing solutions could be used to explicitly model this conformational variability (*e.g.* Burnley *et al.*, 2012 ▸; Levin *et al.*, 2007 ▸).

We argue that models should be built as complete as possible given the data, because high completeness will increase their usefulness to the user community. For instance, simulations of protein-complex formation might improve when the protein structure model is as complete (and correct) as possible: it is better to have a starting experimental conformation available even if supported only by weak electron density, rather than predicting by purely computational methods. Also, the presence of loops in the refinement improves the local-structure quality of the loop surroundings because the added atoms impose better conformational restraints in the structural neighborhood.

In some instances, our methods were unable to build a missing loop in an area with relatively clear electron density. This had to do with either the lack of well aligned homologous loops or the poor geometry of the fitted fragments. In some cases, this could point to register errors (a few residues aligned erroneously onto the sequence). Better comparison between homologous structures, for instance, using tools like *phenix.structure_comparison* (Moriarty *et al.*, 2018 ▸), could be used to identify such regions more reliably; this is not feasible in an automated fashion with the current methods.

**Figure 8:**
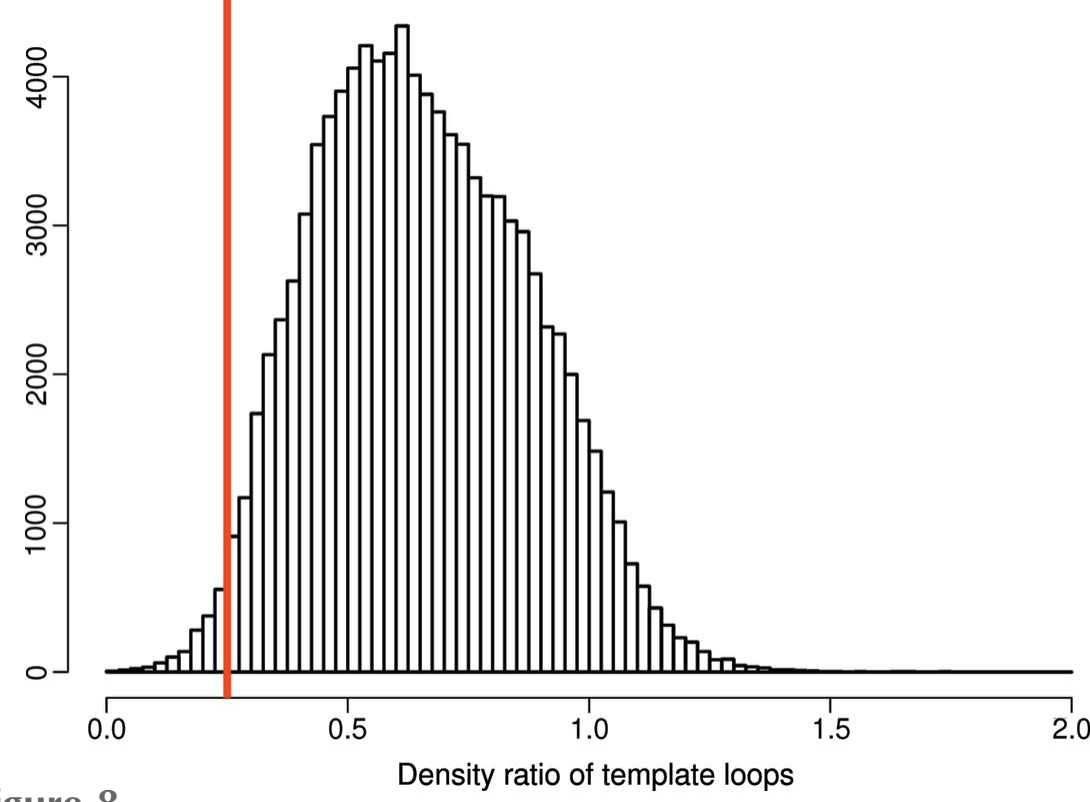
Frequency of specific density ratios of loop backbone *versus* the rest of the backbone for the loops that were used as template from homologous structures. Most template loops have sufficient density and therefore they would also have been built by *Loopwhole*: those loops are to the right side of the red line indicating the density ratio cutoff of 0.25.

The algorithms we have developed to decide whether loops should be kept or not may also be applied to existing parts of protein structure models. We have emphasized in this paper the number of loops that are *not* built in the PDB but may be buildable; however, there may also be cases where crystallographers have modeled loops over-enthusiastically. To estimate the extent of this, we analyzed the distribution of the density ratio between atoms in loops and random atoms in the template structures (the structures where the loops were copied from). This density ratio is better for the template loops than the density threshold cutoff for the newly built loops in most cases (Fig. 8 ▸); this is to be expected because the loops are normally missing precisely because their electron density was not very clear. However, there are also cases where the density fit of the template loop is quite poor; inspection of several cases shows that the majority of these loops are likely to have a correct conformation that is supported by the electron density. However, there are also cases where the loop should not have been modeled.

A possible addition to the methods presented here is building partial loops or expanding the termini. At first, we allowed partial loops to be built by *Loopwhole* if the density fit for that part of the loop was above the density threshold. Although the majority of the 8217 partial loops we built were modeled correctly, building partial loops was not sufficiently reliable to automate. Too often, an amino acid would be modeled into the neighboring density of water molecules or unidentified ligands such as PEG. The same issue would arise for terminal extensions with two additional difficulties. First, the number of residues that may be added is not always clear. Many residues can be missing from the terminus and, since it is unclear *a priori* how far the terminus can be extended, the residues should be added one by one. This is less efficient than loop fitting and therefore likely to cost much more CPU time per added residue. Second, instead of two anchor points on the ends of a missing loop, a terminus only has a single anchor point: the current terminus. The absence of a second anchor point means a drastic loss of information about the general direction in which the residues should be built. Therefore, it is more difficult to detect cases where the expanded fragment is not built into the correct electron density. The combination of these two limitations has kept us from implementing termini extension at present.

New structure models are added to the PDB every week, enriching the set of homologous structure data. The availability of suitable candidates for loop transfer will therefore only increase further, facilitating loop modeling for new structures. The pro-active updating of existing structure models by PDB-REDO ensures that older structure models also benefit from the increased availability of homologs. The original struggle to find a good loop conformation for the first published structure model in a protein family will remain, but it has become a temporary problem; solving the first structure of a protein provides a handle for much future structural research and now in return this future research also provides means to make this first structure more complete. We have clearly demonstrated that the increased availability of homologous data can be used to improve the completeness of protein structure models of the past, the present and the future.

## 5. Availability   

Both the PDB-REDO databank and webserver are hosted on https://pdb-redo.eu. On the webserver, crystallographers can submit their work-in-progress models to run *PDB-REDO* including the new loop-building procedure. The 1990 models from the test set are available through the databank. Existing databank entries are gradually updated to include the loop-building procedure. On the PDB-REDO databank entry pages, registered users can submit an update request to prioritize the update of that entry. Binary executables of *Loopwhole*, *fixDMC* and *tortoize* are available from the website and source code is available on request.

## 6. Related literature   

The following reference is cited in the supporting information for this article: Wang & Dunbrack (2005 ▸).

## Supplementary Material

Supplementary methods and figures.. DOI: 10.1107/S2052252518010552/jt5027sup1.pdf

## Figures and Tables

**Table 1:** Number of built loops and affected structures in the PDB and PDB-REDO databanks. We used 112 385 PDB-REDO entries available in February 2018

Model origin	No. of loops built	No. of entries modified
PDB	22480	10806
PDB-REDO	24962	11571
