# Supplementary material for: Homology-based loop modeling yields more complete crystallographic protein structures
Source: IUCrJ. 2018 Aug 8;5(Pt 5):585–94. doi: 10.1107/S2052252518010552 (PMC6126648; doi:10.1107/S2052252518010552)
Supplement: Supplementary file 1 [file m-05-00585-sup1.pdf]

IUCrJ

Volume 5 (2018)

Supporting information for article:

Homology-based loop modeling yields more complete crystallographic protein structures

Bart van Beusekom, Krista Joosten, Maarten L. Hekkelman, Robbie P. Joosten and Anastassis Perrakis

S1. Supplementary Methods

S1.1. Calculating Ramachandran and rotamer Z-scores

A program called *tortoize* was developed to calculate Z scores for Ramachandran angle quality and rotamer torsion angle quality. The algorithm was implemented in a very similar way to the original implementation (Hooft *et al.*, 1997) but with several small adaptations. Mainly, due to increased availability of (experimental) data it is now possible work with a more reliable dataset, yielding more accurate Z scores. The biggest changes are the more extensive filtering of residues used in the data set and a reduction of the bin size in which residues are scored.

First, residues to analyze have to be selected. We chose to look at protein chains from PDB-REDO entries with a resolution of 2.0 Å or better, an R_{free} of at most 0.25 and a length of at least 40 amino acids. This set was filtered at 70% sequence identity using the Pisces server (Wang & Dunbrack, 2005), yielding a final set of 14,213 protein chains. From this set, 90% was used to collect residue data and 10% to score the protein chain. Residues were selected if i) they have a complete side-chain, ii) they do not have alternate conformations and all atoms are at full occupancy, iii) they are not directly adjacent to a gap or terminus, iv) they are one of the standard 20 amino acids or seleno-methionine (MSE), v) the ω angle is not distorted (i.e. more than 45 degrees off from 0 or 180) and vi) the density metrics are good: RSCC ≥ 0.95 and RSR ≤ 0.12 according to EDSTATS (Tickle, 2012).

The data was then separated into categories. For both Ramachandran and rotamer validation, data are divided per amino acid and per secondary structure type (α-helix, β-strand and other). For Ramachandran angle validation, four extra categories are required: one for *cis*-proline and three to accommodate deviations for pre-proline residues, which are split in Gly-prePro, Ile/Val-prePro and all other pre-proline residues. These last four categories are not split on secondary structure because the amount of available data is insufficient. Hence, there is a total of 64 categories for Ramachandran angle validation.

Bins were defined in the 2D space with a spacing of 2°. The bins were circular with a radius of √2° so that all data points are included at least once. The number of data points of each bin was then counted and expected values and standard deviations were computed exactly as described (Hooft *et al.*, 1997). The remaining 10% of protein chains in the test set were then scored against these data files to obtain a measure of how well an average high-quality protein scores, again exactly as described (Hooft *et al.*, 1997).

In the implementation in *Loopwhole*, the relevant data files are read for the residues in the loop and the Z scores per residue are computed from those data. Averaging the Z scores per residue gives the Z score of the loop.

References

Hooft, R. W., Sander, C. & Vriend, G. (1997). *Comput. Appl. Biosci.* **13**, 425–430.

Tickle, I. J. (2012). *Acta Crystallogr. D Biol. Crystallogr.* **68**, 454–467.

Wang, G. & Dunbrack, R. L., Jr (2005). *Nucleic Acids Res.* **33**, W94–W98.

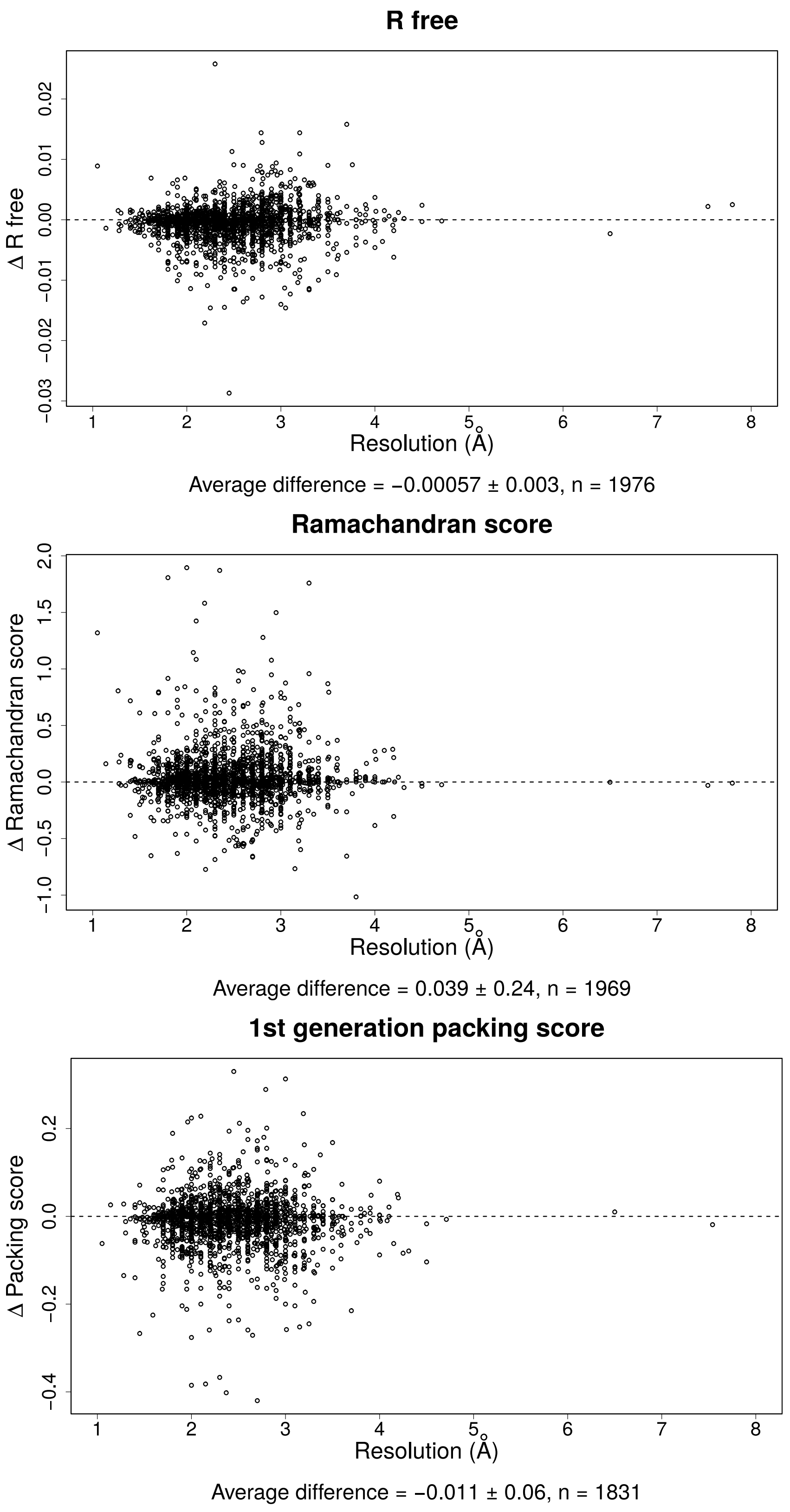

Figure S1 Impact of loop building on standard validation metrics.

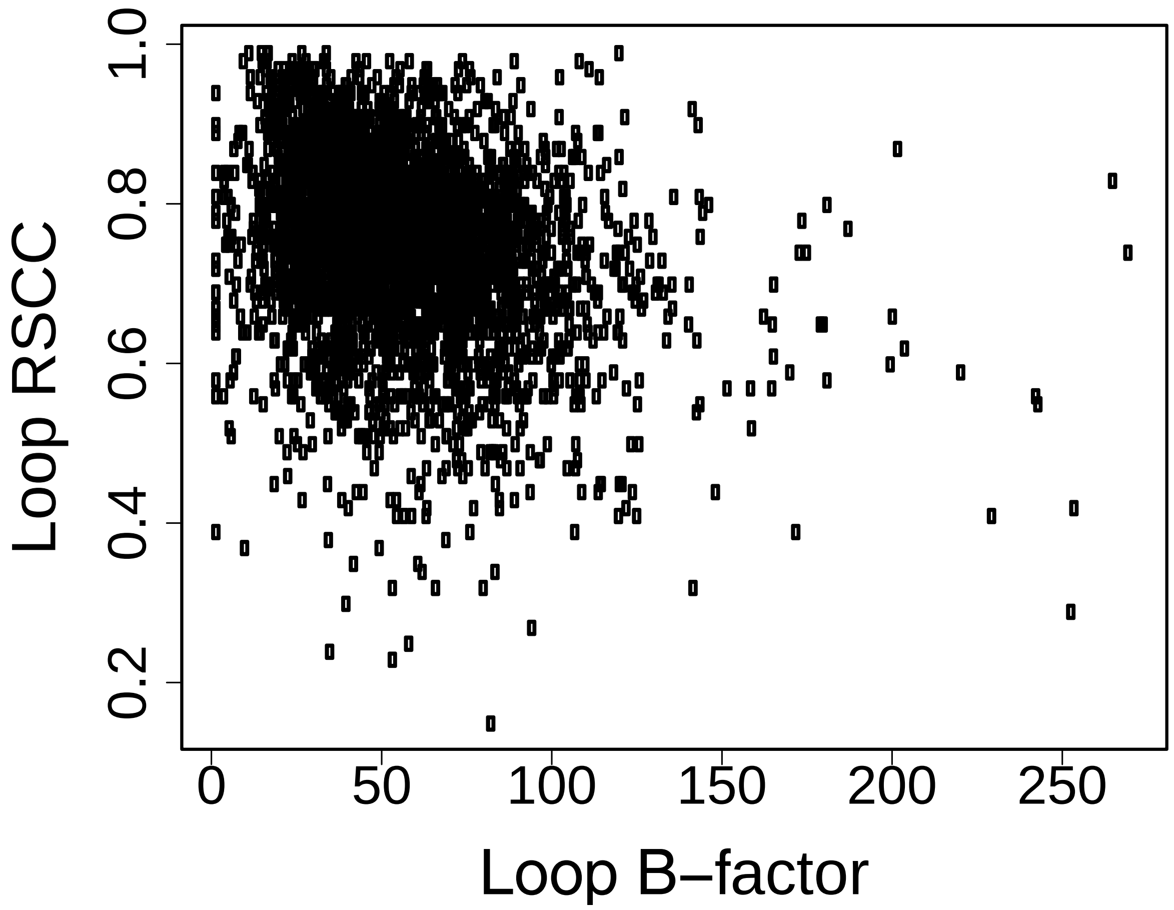

Figure S2 The average B-factor versus the RSCC for all loops built in the PDB-REDO test set.
